# Maternal High-Fat Feeding Affects the Liver and Thymus Metabolic Axis in the Offspring and Some Effects Are Attenuated by Maternal Diet Normalization in a Minipig Model

**DOI:** 10.3390/metabo11120800

**Published:** 2021-11-26

**Authors:** Federica La Rosa, Letizia Guiducci, Maria Angela Guzzardi, Andrea Cacciato Insilla, Silvia Burchielli, Maurizia Rossana Brunetto, Ferruccio Bonino, Daniela Campani, Patricia Iozzo

**Affiliations:** 1 Institute of Clinical Physiology, National Research Council (CNR), 56124 Pisa, Italy; larosa.fed@gmail.com (F.L.R.); letiziag@ifc.cnr.it (L.G.); m.guzzardi@ifc.cnr.it (M.A.G.); 2 Department of Surgical, Medical, Molecular Pathology and Critical Care Medicine, Division of Pathology, Pisa University Hospital, 56124 Pisa, Italy; andrea.cacciatoinsilla@gmail.com (A.C.I.); daniela.campani@med.unipi.it (D.C.); 3 Gabriele Monasterio Foundation, 56124 Pisa, Italy; silvia.burchielli@ftgm.it; 4 Department of Clinical and Experimental Medicine, University of Pisa, 56124 Pisa, Italy; maurizia.brunetto@unipi.it; 5 Hepatology Unit, Department of Medical Specialties, Laboratory of Molecular Genetics and Pathology of Hepatitis Viruses, Pisa University Hospital, 56124 Pisa, Italy; 6 Institute of Biostructure and Bioimaging (IBB), National Research Council (CNR), 80145 Napoli, Italy; ferruccio.bonino@unipi.it

**Keywords:** fetal programming, maternal high-fat diet feeding, liver, thymus, positron emission tomography, minipig, insulin resistance

## Abstract

Maternal high-fat diet (HFD) affects metabolic and immune development. We aimed to characterize the effects of maternal HFD, and the subsequent diet-normalization of the mothers during a second pregnancy, on the liver and thymus metabolism in their offspring, in minipigs. Offspring born to high-fat (HFD) and normal diet (ND) fed mothers were studied at week 1 and months 1, 6, 12 of life. Liver and thymus glucose uptake (GU) was measured with positron emission tomography during hyperinsulinemic-isoglycemia. Histological analyses were performed to quantify liver steatosis, inflammation, and hepatic hematopoietic niches (HHN), and thymocyte size and density in a subset. The protocol was repeated after maternal-diet-normalization in the HFD group. At one week, HFD_off_ were characterized by hyperglycemia, hyperinsulinemia, severe insulin resistance (IR), and high liver and thymus GU, associating with thymocyte size and density, with elevated weight-gain, liver IR, and steatosis in the first 6 months of life. Maternal diet normalization reversed thymus and liver hypermetabolism, and increased HHN at one week. It also normalized systemic insulin-sensitivity and liver fat content at all ages. Instead, weight-gain excess, hyperglycemia, and hepatic IR were still observed at 1 month, i.e., end-lactation. We conclude that intra-uterine HFD exposure leads to time-changing metabolic and immune-correlated abnormalities. Maternal diet-normalization reversed most of the effects in the offspring.

## 1. Introduction

Obesity, commonly due to the intake of high-fat, energy-rich foods, is a common cause of chronic diseases and metabolic dysregulation [1]. In the last decades, its impact has increased worldwide, involving a large number of women of childbearing age, with negative health consequences for mothers and offspring [2,3]. The intrauterine metabolic environment can permanently program the development and physiology of the offspring with short- and long-term health implications [4,5]. We previously reported that high-fat diet (HFD) consumption during pregnancy affects the brain glucose metabolism and myocardial function in the offspring [2,3], but the metabolic impact on liver and immune organs, from birth to late adulthood, has not been elucidated. During fetal and early post-natal life [6,7], the liver generates blood and immune cells in specialized areas called niches, providing a supportive microenvironment for highly proliferative hematopoietic stem cells [8], and cooperates with the thymus in T lymphocyte production and maturation [9,10,11,12,13]. Maternal HFD was shown to compromise the hematopoietic compartment in the fetal liver [14]. The liver is the main center for glucometabolic control, and glucose is the primary substrate used by thymocytes and T lymphocytes, whose metabolic alterations can associate with immune intolerance and metabolic syndrome, since fetal life [15,16,17,18]. Liver insulin resistance (IR) and glucose dysmetabolism in response to nutritional stress are reported in the fetus of HFD mothers, increasing the risk of fatty liver disease [19], which may affect the development of hepatic lymphocytes [13,20]. However, the occurrence of fatty and inflammatory liver disease in the offspring of HFD mothers remains controversial [21,22]. The current study was undertaken to examine the hypothesis that maternal HFD has a life-lasting impact on hepatic glucose uptake (GU), insulin sensitivity, and steatosis/steatohepatitis in the offspring. Moreover, the scope of our study was to examine the potential benefits of dietary intervention on mothers before conception, namely whether weight loss in HFD mothers before a second pregnancy would reduce abnormalities in insulin sensitivity and organ metabolism, as expected in offspring of HFD mothers. We also hypothesized that the liver and thymus metabolism, and their hematopoietic cellularity, would show associations, providing mechanistic insight underlying their cross-talk. The study was conducted in minipigs, as a suitable model to address the human metabolism and obesity [23]. We used positron emission tomography (PET) and 2-[18F]-fluoro-2-deoxyglucose ([18F]-FDG) during hyperinsulinemic-isoglycemia to measure the liver and thymus glucose metabolism in vivo, and ex vivo histology to assess hepatic fatty and hematopoietic infiltration and inflammation, and tested correlations between tissue metabolism and cellularity.

## 2. Results

**Figure 1:**
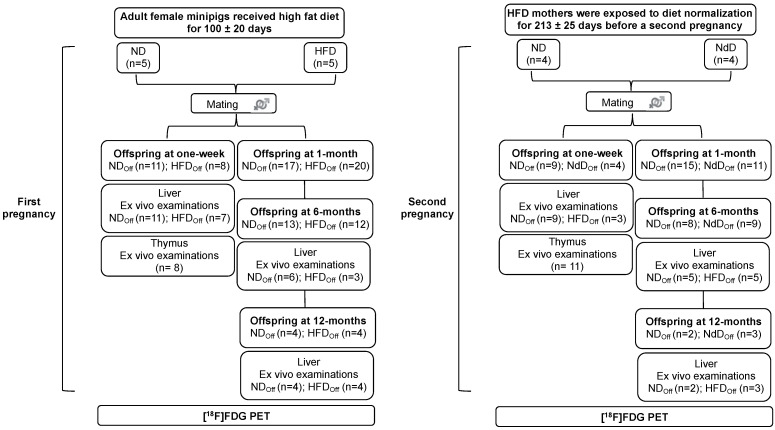
Study design. The study was carried out in the offspring of adult female minipigs fed a normal (ND) or high-fat diet (HFD) at one week and at 1, 6, and 12 months of age; then, HFD mothers were returned to normal diet (NdD), and the offspring from a second pregnancy were studied. [18F]-FDG-PET was used to quantify liver and thymus metabolism under hyperinsulinemic-isoglycemia. Offspring were euthanized, and liver and thymus gland samples collected for ex vivo histological analyses to support the understanding of metabolic results.

The study was carried out in adult female primiparous minipigs, undergoing two consecutive pregnancies, and their offspring. Mothers in the first pregnancy received a normal diet (ND) or high fat diet (HFD) and were studied before and during the gestations. After weaning of the pups, HFD mothers were exposed to diet normalization (NdD) and then restudied in a second pregnancy, in parallel to control mothers (still on ND). Groups of offspring born to mothers undergoing high-fat diet (HFD_off_), normal diet (ND_off_), and diet normalization (NdD_off_) were studied at 1 week, and 1, 6, and 12 months, by [18F]-FDG PET imaging during hyperinsulinemic-isoglyaemia, and liver and thymus histology, according to the flowchart given in Figure 1.

### 2.1. Maternal Body Weight and Glucose Metabolism

Duration of gestation was similar in all groups. By design, HFD mothers were heavier, and had a larger waist circumference than ND mothers before and during pregnancy (Table 1) in the first, but not in the second, pregnancy. ND mothers experienced weight gain between the end of the first pregnancy and the beginning of the next, consistent with previous observations in rodent studies addressing multiparity [24]. In contrast, in HFD mothers, diet normalization resulted in a weight loss of 7.0 ± 2.7 kg, corresponding to ~1 kg per month (=12% reduction in seven months). No significant difference in fasting glycemia and whole-body insulin sensitivity was observed, though an expected tendency towards IR in HFD mothers was present (*p* = 0.1). Liver GU was similar in mothers before and during the first pregnancy and before the second gestation, whereas a downward tendency was observed during the second pregnancy (*p* = 0.06). ND mothers underwent a significant increase in liver GU during the first pregnancy compared to pre-gestational values (*p* = 0.048), whereas NdD was the only maternal group showing a decline in liver GU from pre-pregnancy to pregnancy values (*p* = 0.03).

### 2.2. Body Weight and Systemic Metabolism in the Offspring

**Figure 2:**
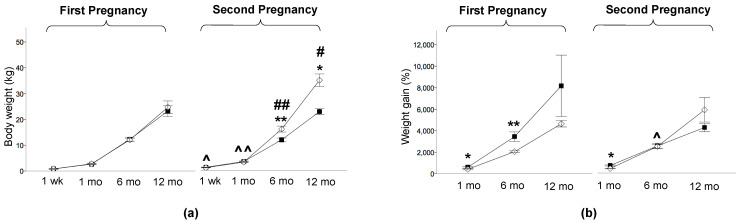
Body weight and weight gain. (**a**) Body weight at one week, 1 month, 6 months, and 12 months of age in the offspring of HFD and ND mothers, after the first and second pregnancy, and (**b**) weight gain in the first month, 6 months, and 12 months of life. Black squares: offspring of HFD mothers in first pregnancy and NdD mothers in second pregnancy after diet normalization; white diamonds: offspring of ND mothers. Values are means ± SEM. * *p* < 0.05, ** *p* < 0.01 vs. age-matched control within each pregnancy; # *p* ≤ 0.05, ## *p* < 0.01 ND_off_ in second pregnancy vs. respective ND_off_ in the first; ^ *p* ≤ 0.05, ^^ *p* < 0.01 NdD_off_ in second pregnancy vs. respective HFD_off_ in the first.

**Figure 3:**
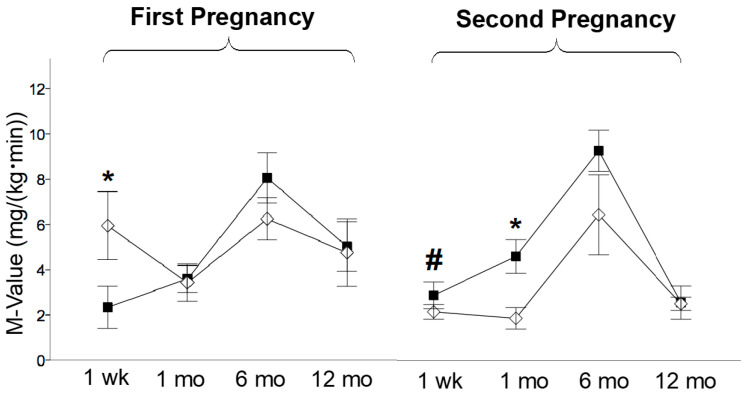
Whole body insulin sensitivity. M-value at one week, 1 month, 6 months, and 12 months of age in the offspring of HFD and ND mothers, after the first and second pregnancy. Black squares: offspring of HFD mothers in first pregnancy and NdD mothers in second pregnancy after diet normalization; white diamonds: offspring of ND mothers. Values are means ± SEM. * *p* < 0.05 vs. age-matched control within each pregnancy; # *p* ≤ 0.05 ND_off_ in second pregnancy vs. respective ND_off_ in the first.

The offspring studied at one week were weighted and sacrificed on the same day of the PET study. The offspring studied at 1, 6, and 12 months of life were weighed on the day after delivery and on the day of the PET study to compute weight gain, which was expressed as a %. HFD_off_ showed a low birth weight in the first (0.421 ± 0.029 vs. 0.551 ± 0.012 kg, *p* < 0.0001, HFD_off_ vs. ND_off_) and in the second pregnancy, after maternal diet normalization (0.468 ± 0.025 vs. 0.621 ± 0.013 kg, *p* < 0.0001, NdD_off_ vs. ND_off_), and body weight increased in ND_off_ in the second compared to the first pregnancy (0.621 ± 0.013 vs. 0.551 ± 0.012 kg, *p* < 0.0001). No significant body weight difference was observed between offspring groups within each pregnancy at the time of PET imaging, but offspring born to the second pregnancy were heavier than those born to the first pregnancy (NdD_off_ at 1 week and 1 month, and ND_off_ at 6 and 12 months, leading to higher adulthood weight in ND_off_ than NdD_off_) (Figure 2a). Weight gain was greater in HFD_off_ (from birth to 1 and 6 months) and NdD_off_ (from birth to 1 month) compared to ND_off_, whereas it was lower in 6-month-old NdD_off_ than HFD_off_ (Figure 2b). Waist circumference was larger in the second than the first pregnancy in both groups (Table 2), but the increase was transient in NdD_off_ whereas it lasted until late adulthood in ND_off_; consequently, NdD_off_ had lower values than respective ND_off_. Glycemia (Table 2) tended to be higher in HFD_off_ at 1 week (significant only before sex-adjustment), and was significantly higher in NdD_off_ at 1 month than respective ND_off_. Insulin levels tended to be higher in HFD_off_, and were lower at 1 month (*p* < 0.01) in NdD_off_, compared to respective controls (Table 2). Whole-body insulin sensitivity (M-value) showed remarkable IR at 1 week in HFD_off_ compared to ND_off_, and in 1-month-old ND_off_ born to the second pregnancy compared to NdD_off_, with no difference thereafter (Figure 3).

### 2.3. Liver and Thymus Glucose Uptake in the Offspring

**Figure 4:**
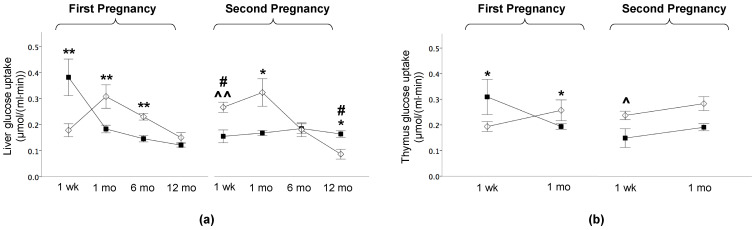
Liver and thymus glucose uptake in the offspring. (**a**) Glucose uptake in liver at one week and 1 month, 6 months, and 12 months of age in the offspring of HFD and ND mothers, after the first and second pregnancy. (**b**) Thymus glucose uptake was measured at one week and 1 month of age, i.e., before gland involution. Black squares: offspring of HFD mothers in first pregnancy and NdD mothers in second pregnancy after diet normalization; white diamonds: offspring of ND mothers. Values are means ± SEM. * *p* < 0.05, ** *p* < 0.01 vs. age-matched control within each pregnancy; # *p* ≤ 0.05 ND_off_ in second pregnancy vs. respective ND_off_ in the first; ^ *p* ≤ 0.05, ^^ *p* ≤ 0.01 NdD_off_ in second pregnancy vs. respective HFD_off_ in the first.

At 1 week, liver GU was 100% greater in HFD_off_ than ND_off_ (*p* < 0.001) (Figure 4a), and this difference was not observed in the second pregnancy due to a significant reduction in NdD_off_ and a significant increase in ND_off_ in the second compared to the first pregnancy. At 1 and 6 months, HFD_off_ in the first pregnancy showed reduced insulin-mediated hepatic GU, i.e., liver IR, persisting in NdD_off_ only at 1 month in the second gestation, corresponding to end lactation (Figure 4a). Thymus GU was higher at 1 week and lower at 1 month in HFD_off_ than ND_off_ in the first pregnancy, and this difference was attenuated in offspring born to the second gestation (*p* = 0.06) (Figure 4b).

### 2.4. Liver Histology, Transaminases, Triglycerides

**Figure 5:**
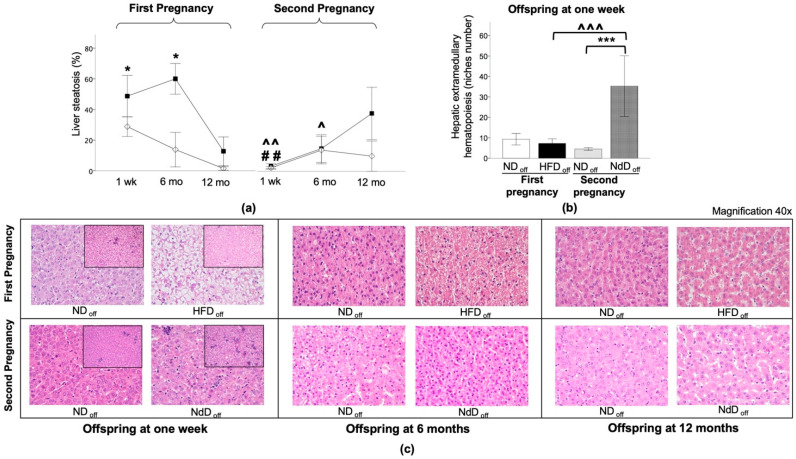
Liver histology in the offspring. (**a**) Liver steatosis at one week, 6 months, and 12 months in the offspring of HFD and ND, after the first and second pregnancy. (**b**) Hepatic extramedullary hematopoiesis (niches number) at one week. Black squares: offspring of HFD mothers in first pregnancy and NdD mothers in second pregnancy after diet normalization; white diamond: offspring of ND mothers. Values are means ± SEM. * *p* < 0.05, *** *p* < 0.001 vs. age-matched control within each pregnancy; ## *p* < 0.01 ND_off_ in second pregnancy vs. respective ND_off_ in the first; ^ *p* ≤ 0.05, ^^ *p* ≤ 0.01, ^^^ *p* ≤ 0.001 NdD_off_ in second pregnancy vs. respective HFD_off_ in the first pregnancy. (**c**) Representative liver histological sections of offspring at one week, 6, and 12 months of HFD and ND mothers after the first (top) and second (bottom) pregnancy (40× magnification) are presented. Representative examples of histological sections of hepatic extramedullary hematopoiesis niches (small image, 20× magnification) of the liver in the offspring at one week of HFD and ND mothers, after the first and second pregnancy are shown.

HFD_off_ showed significant liver steatosis at 1 week and 6 months of age (Figure 5a), with 100% HFD_off_ vs. 17% ND_off_ having a diagnosis of fatty liver disease (*p* = 0.045) (Table 3), and high triglyceride (TG) levels (25 ± 6 vs. 11 ± 2 mg/dL, *p* = 0.017). Liver steatosis was normalized in NdD_off_ (*p* = 0.005 and *p* = 0.016, compared to HFD_off_ at 1 week and 6 months), together with TG levels (28 ± 4 vs. 21 ± 5 mg/dL, ns vs. ND_off_). No significant difference was observed between groups at 12 months (Figure 5a), or in other features of liver injury including lobular or portal inflammation, hepatocyte ballooning degeneration, and fibrosis between groups (Table 3). Extramedullary hematopoiesis niche numbers showed a slight reduction in 1-week-old HFD_off_, and a significant increase in NdD_off_ compared to ND_off_ (*p* = 0.01) and HFD_off_ (*p* = 0.03) (Figure 5b).

### 2.5. Correlations

**Figure 6:**
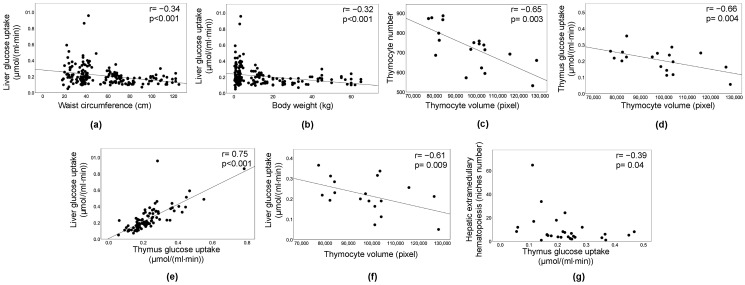
Correlations. Inverse relationship between liver glucose uptake and waist circumference (**a**) or body weight (**b**). The volume of thymocytes was expectedly correlated with their number per unit area (**c**), and with thymus glucose uptake (**d**). Liver and thymus glucose uptake rates were correlated (**e**), liver glucose uptake was also predictive of lower thymocyte volume (**f**). A greater number of hematopoietic niches in the liver was associated with thymus insulin resistance (**g**); (**a**–**g**) involve normally distributed variables, and therefore a trend line is shown and sex adjustment performed (partial correlation analysis), different from (**g**) (Spearman’s regression coefficient).

Both univariate and sex-adjusted associations (Figure 6) were examined, showing consistent levels of significance. An inverse relationship was observed between liver GU and waist circumference (Figure 6a) or body weight (Figure 6b), indicating that higher weight and abdominal obesity resulted in lower liver GU and greater hepatic IR in the whole population. Thymus histology showed that thymocyte volume was inversely related to cell density (Figure 6c) and tissue GU (Figure 6d), indicating that higher GU in the thymus reflected numerous small thymocytes, whereas thymus IR reflected fewer larger thymocytes. Liver and thymus GU rates were correlated (Figure 6e). Liver GU was also predictive of lower thymocyte volume (Figure 6f), resulting in greater density. In addition, low hematopoietic niche numbers in the liver were associated with thymus hypermetabolism (Figure 6g).

## 3. Discussion

The offspring of obese mothers have an increased risk of developing IR, metabolic disorders, and immune dysfunction [14,25,26,27]. This study is part of a large project focused on cardiometabolic consequences of maternal obesity [2,3,28] and here we report our advancement of knowledge concerning the liver and thymus. The original design did not address the thymus. In the course of image analyses, we observed an elevated GU in the thymus, and started collecting thymus tissue samples. Thus, the main study limitation is that thymus histology was available in 19 cases, and could only be used in correlations, lacking sufficient power for group comparisons. Notably, however, PET images were available in all animals and provide the first evidence of compromised thymus metabolism in the offspring born to obese mothers, as determined by in vivo imaging within the first month of life, before age-dependent thymic involution [29].

HFD mothers displayed the expected increase in body weight and waist circumference [30], accompanied by a tendency towards systemic IR. The first gestation was accompanied by a significant increase in liver GU in ND mothers compared to pre-pregnancy values, in line with the notion that in the normal situation, glucose requirements of the gravid uterus are increased [31], together with post-prandial hepatic glycogen stores for an adequate glucose supply to maternal and fetal tissues [32]. HFD and NdD mothers lacked this upregulation, showing no change or downregulation in liver GU. The response was also blunted in the second gestation in ND mothers, likely due to the weight gain associated with multiparity. We suggest that the maternal liver responds to the sensing of glucose levels in the developing offspring, modulating the need for hepatic GU and storage.

Maternal HFD resulted in normal body weight in the offspring at one week of life, and lower body weight at birth, but higher weight gain through adulthood compared to ND_off_. Our data are in agreement with, e.g., Jungheim et al. [33], showing that exposure to diet-induced maternal obesity results in smaller offspring at birth, undergoing later catch-up growth. However, the birth weight outcome of maternal HFD is controversial [34], and the HFD used in mice is more standardized than that observed in humans. In the second pregnancy, after diet normalization in HFD mothers, birth weight was still low, and weight gain was still elevated between birth and 1 month (but not 6 months) in HFD_off_. Catch-up growth is recognized as an unfavorable condition [35], associated with later metabolic conditions in humans. Our data suggest that maternal dietary restriction did not correct the initial excess in weight gain at 1-month (end-lactation), but resolved the subsequent weight gain through adulthood. Results align with the observation of lower obesity risk in children and young adults born after maternal weight loss [36]. Of further note, body weight was elevated at birth and in adult ND_off_ born to the second pregnancy, supporting the concept that maternal pre-pregnancy weight gain reflects on the adult offspring, independent of diet composition. Interestingly, Rebholz et al. showed that body weight was increased in offspring of multiparous dams, leading to metabolic dysfunction, compared to offspring of primiparous mothers [24]. They comment that weight gain retention between consecutive pregnancies is common in women, with a risk of multiparity-induced obesity.

Glycemia was high at 1 week in HFD_off_, in agreement with prior evidence in several animal models [18,37]. Similar to weight gain, glucose levels were elevated at end-lactation in NdD_off_ born to diet-restricted mothers. These observations implicate that hyperglycemia at birth (as seen in HFD_off_) results from intrauterine overfeeding, whereas hyperglycemia in later life-stages may depend more directly on post-natal weight gain, with a possible influence of lactation. Baseline insulin levels tended to be elevated in 1-week HFD_off_, consistent with their pronounced systemic IR, in agreement with human evidence showing IR at birth in offspring of obese women [38]. In the second pregnancy, hyperinsulinemia and systemic IR were evident at 1 month in ND_off_, again suggesting that maternal weight gain between subsequent pregnancies results in similar offspring outcomes compared to maternal HFD, consistent with the observation of metabolic dysfunction caused by maternal multiparity in mice [24].

Our results show that HFD_off_ are born with hepatic glucose hypermetabolism, likely depending on their hyperglycemic state. From previous studies, we know that GU in the liver promotes lipid accumulation [39]. In fact, liver lipid accumulation was observed from birth to adult age in our HFD_off_, in which the frequency of intra-hepatocyte triglyceride vesicles was 4–5 folds higher than in ND_off_. Results are consistent with evidence in adult mice presenting an increase in hepatic lipid content and molecular IR [19], or hepatic mitochondrial dysmetabolism and lipogenesis [22], followed by significant liver IR [40]. In line with those studies, we observed severe hepatic IR ensuing at 1 and 6 months. Several studies indicate that maternal HFD per se is an insufficient determinant of hepatic steatosis or steatohepatitis unless associated with HFD in the offspring [21,22,41,42]. In support of this, we could not identify significant inflammatory or fibrotic damage in this group, which might require post-natal HFD, and our HFD_off_ underwent faster weight gain (compared to ND_off_) which may contribute to the increase in liver lipids observed in adult life. The negative association linking liver GU and body weight or waist circumference in this study further supports an involvement of visceral adiposity to reinforce hepatic lipid accumulation and IR. Notably, NdD_off_ born after maternal diet normalization showed transient hepatic IR at 1 month, followed by a normal liver metabolism and a significant reduction in liver lipids compared to HFD_off_, resulting in a normal content. This is consistent with the attenuation of fetal hepatic steatosis following a healthy maternal diet [43], and documents that in HFD mothers, a few months of dietary normalization in advance of a second pregnancy can lead to a metabolically healthy liver in the adult offspring. It is also important to note that most persisting metabolic abnormalities (weight gain, hyperglycemia, liver IR) in NdD_off_ were seen only at the age of 1 month, i.e., end of lactation, suggesting that early post-natal nutrition might still be suboptimal after weight normalization.

Our data showed a slight non-significant reduction in the number of hepatic extramedullary hematopoiesis niches (HHN) in HFD_off_ and a significant increase in niche numbers in NdD_off_, together suggesting an impact of maternal diet on hepatic hematopoiesis. HHN influence the exposure to the multitude of new antigenic encounters in early life, when different immune cells arise from distinct waves of hematopoietic stem cells within HHN in the critical transition from a relatively sterile fetal to a new microbial environment, educating the developing immune system. Thus, an altered and reduced HHN number might hamper the imprinting of the immune system and increase disease-risks throughout life, including hepatic steatosis and inflammation triggered by e.g., microbial products reaching the liver. Future studies of the thymus “hepato-enteric” immune axis in these animal models might provide new insights into the pathogenesis of many inflammatory diseases affecting both anatomical sites [44]. An interesting study in mice showed that chronic HFD compromises fetal liver hematopoiesis and cellularity, with a defective content of hematopoietic stem and progenitor cells and an increase in differentiated blood cells in the liver, which were prematurely released from the fetal liver to bone marrow [14], and the thymus gland for maturation and differentiation of T lymphocytes [14,20]. In line with this, our results showed that HFD_off_ were born with high thymic-insulin-mediated GU, correlating with smaller and more numerous thymocytes. These features were related to greater liver GU and fewer HHN, consistent with the above concept of a premature cellular overload to the thymus. Our results also reinforce in vitro evidence, showing that glucose levels in the incubating medium stimulate thymocyte glucose uptake in a dose-response fashion [16], and that HFD leads to increased thymocyte numbers [45]. Though insulin and hyperglycemia are important regulators of the peripheral lymphocyte metabolism [46,47,48], unfortunately peripheral lymphocytes were not collected. Overall, the previous literature and our hepatic and thymic findings lead us to speculate that a hypermetabolic liver in HFD_off_ accelerates progenitor maturation and dislocation to the thymus at newborn age.

## 4. Materials and Methods

### 4.1. Animal Model and Study Design

The study (Figure 1) was carried out in offspring of female minipigs, undergoing two consecutive pregnancies, after mating with the same male minipig.

First pregnancy. Adult female minipigs received a HFD (+740 kcal from fat daily for 10 weeks, +370 kcal from fat daily thereafter) (n = 5) or a normal diet (ND, ~1850 kcal daily) (n = 5) throughout gestation and lactation. Fresh water was provided ad libitum. After 100 ± 20 days, mothers were mated and allowed to deliver spontaneously. After weaning, offspring were fed a ND, and groups were studied (by PET) within 1 week of birth (n = 19, 11/8 females/males) and at 1 month (n = 37, 14/23 f/m, end of lactating period), 6 months (n = 25, 7/18 f/m), and 12 months (n = 8, 1/7 f/m), the latter representing early and late adulthood, respectively. All animals were studied at 1 week, and parts of the following groups were euthanized by anesthesia overdose after imaging for tissue collection. 

Second pregnancy. After lactation, HFD mothers were exposed to diet normalization (NdD, n = 4) for 213 ± 25 days before a second pregnancy. The control group (ND, n = 4) was maintained under the same standard diet. Offspring were studied at 1 week (n = 13, 5/8 f/m), 1 month (n = 26, 10/16 f/m), 6 months (n = 17. 6/11 f/m), and 12 months (n = 5, 1/4 f/m) of age undergoing the same measurements described for the first pregnancy.

[18F]-FDG-PET imaging was used to measure liver and thymus gland GU during isoglycemic-hyperinsulinemia. Liver biopsies were collected in most animals, whereas thymus biopsies were taken in a subset of minipigs at 1 week of age to measure thymocyte size and density. The experimental protocol was conducted in accordance with the D.L.116/92 implementation of European Economic Community directive 609/86 regarding the protection of animals used for experimental and other scientific purposes.

### 4.2. [18F]-FDG PET

After an overnight fast, anesthesia was induced with tiletamine-zolazepam (10 mg/kg Zoletil; Virbac Laboratories, Carros, France) and chlorpromazine (1 mg/kg, Largactil; Sanofi Aventis, Gentilly-Cedex, France), and maintained by an infusion of zolazepam (2 mg⋅kg^−1^⋅h^−1^ Zoletil). After body weight and basal glycemia determination, catheters were placed into one ear vein for glucose, insulin, and tracer administration, and in the contralateral ear vein for anesthesia. We carefully catheterized only superficial veins, and the amount of blood available was limited. We privileged glucose measurements during the clamp, whereas insulin was determined by enzymatic assay (Architect i1000_SR_, Abbott Laboratories, Chicago, IL, USA) wherever possible before or after the study, once insulin levels had returned to proxy-fasting values (n = 23 offspring at 1 week, n = 22 at 1 month, n = 34 at 6 months, and n = 12 at 12 months of age). Animals were positioned in the gantry of an ECAT HR+ tomograph (Siemens CTI, Knoxville, TN, USA), and a continuous infusion of insulin (1 mU⋅min^−1^⋅kg^−1^) was started. Isoglycemia was adopted during the clamp to reflect daily-life glycemic conditions, and maintained by infusing a 20% glucose solution, adjusted according to frequent blood glucose monitoring by glucometer (OneTouch, Johnson & Johnson Services, Medical SpA, Pomezia, Italy). A transmission scan was performed to correct subsequent emission data for photon attenuation. At t = 45 min, [18F]-FDG was injected, and a 30 min dynamic acquisition of the thoracic and upper abdominal region was carried out. Sinograms were corrected for dead time, decay, and photon attenuation and reconstructed by standard algorithms. Images were analyzed with VINCI software (Vinci64 v4.03, Köln, Germany). Regions of interest were drawn on images corresponding to the left ventricular chamber of the heart (blood input function), and the liver and thymus gland, to obtain respective [18F]-FDG time-activity curves (kBq/mL). Tissue activity in the late frames was divided by the integrated blood activity to quantify the fractional uptake rate of [18F]-FDG (min^−1^) in the liver and thymus, which was multiplied by steady-state glycemia (μmol/mL) to obtain respective GU rates (μmol⋅mL^−1^⋅min^−1^) [49]. Whole body insulin sensitivity (M-value) was measured during the clamp, as described [50].

### 4.3. Histological Analysis

Histological Analysis of Liver. Liver samples were dissected, fixed in 10% neutral buffered formalin for 24 h, and dehydrated in ethanol. Liver biopsies were processed and included in paraffin using the Donatello Diapath automatic tissue processor (Martinengo, Bergamo, Italy), sliced (HistoCore Autocut, Leica BioSystems microtome) with a thickness of 2 μm, and stained with hematoxylin and eosin using the automated Dako CoverStainer (Santa Clara, CA, USA). Each section was documented at 20× and 40× magnification by using the Nikon Eclipse E600 microscope and connected with a Nikon Y-TV55 digital camera and NIS-ElementsD Ver5.20.00 Imaging Software (Nikon Corporation, Shinagawa Intercity Tower C, 2-15-3, Konan, Minato-ku, Tokyo 108-6290, Japan). The analyses were adapted from the method of Kleiner et al. [51], addressing microcirculation (portal, central vein, sinusoidal dilatation), fibrosis (portal, perisinusoidal, perivenular), portal inflammation, and lobular damage (micro- and macrovesicular steatosis, lobular inflammation, i.e., foci number at 20×; ballooning degeneration; glycogenated nuclei). In addition, the presence of extramedullary hepatic hematopoiesis and the number of niches were examined at 20× magnification in 1-week-old minipigs. All parameters were quantified on a categorical yes/no basis; steatosis was also expressed as the % of cells affected (0 ≤ 5% affected cells, 1 = 6–33% affected cells, 2 = 34–66% affected cells, 3 ≥ 67% affected cells), and lobular inflammation was stratified according to severity grades, based on the foci number at 20× magnification (1 = one focus, 2 = two-four foci, 3 ≥ four foci) and ballooning degeneration (few cells = 1, many cells = 2).

Histological Analysis of Thymus. Thymocyte number and size were assessed in 19 available samples. These were fixed in 10% neutral buffered formalin and then dehydrated, included in paraffin (Bio-Optica, Milano, Italy), sliced (Microm HM 330) with a thickness of 3 μm, and stained with hematoxylin and eosin. Each section was documented at 100× magnification using an Axioskop optical microscope connected with an AxioCam MRc5 color-camera and AxioVision analysis software (Carl Zeiss, Oberkochen, Germany). Three histological images for each animal were selected and analyzed with the ImageJ software (ImageJ 1.49v–Wayne Rasband, National Institutes of Health, Bethesda, MD, USA) by measuring thymocyte numbers in a given area and cell diameters in 54 thymocytes per animal. Cell volumes were estimated by assuming a spherical shape, since diameters were similar.

### 4.4. Statistical Analyses

Data are presented as means ± SEM. Statistical analyses were performed using the IBM SPSS Statistics 22.0 software package (SPSS, Chicago, IL, USA). The Shapiro–Wilk test, with a 95% confidence range, was used to evaluate the normality of the data distribution. The Mann–Whitney test was used to analyze weight gain (%) and hematopoiesis niche numbers. ANOVA and ANCOVA (adjusting for sex dimorphism [38,52,53]) with post hoc Fisher LSD (in offspring) or *t*-tests (in mothers) were applied to analyze the other variables, comparing age-matched groups (within and between pregnancies). Sex-adjusted (ANCOVA) statistical results are given in Results, Figures, and Tables, which did not abolish significance (vs. ANOVA), with the exception of 1-week glycemia, as reported (Results). The Chi-square test was applied to compare the distribution of categorical variables among groups. Pearson or Spearman’s regression coefficients were used according to variable distributions, and a partial correlation analysis was performed to adjust for sex. Statistical significance was set at a *p* value ≤ 0.05.

## 5. Conclusions

Our data document that maternal obesity leads to time-changing liver and thymus metabolic dysfunction in minipigs. Lipid accumulation and glucose dysmetabolism in the liver seemed due to the combined action of in-utero HFD exposure and post-natal weight gain. Our results support the concept of a cross-talk between the liver and thymus in the first life-days. Dietary normalization in HFD mothers improved systemic IR, steatosis, and tissue metabolism in newborns and adults. Hyperglycemia, weight gain excess, and liver IR were still observed, limited to the first life-month of lactation. A more prolonged or restrictive diet between subsequent pregnancies might be more fully effective. Overall, this study underscores the effectiveness of maternal preconception dietary control, even for a short period. Considering that dietary restriction is a challenging target, another potential outcome is that post-natal prevention of weight gain and hyperglycemia may confer sufficient protection from life-long consequences of an adverse in-utero nutritional environment. Furthermore, future studies of the “thymus-hepato-enteric” immune-axis might provide new insights into the pathogenesis of diseases affecting these anatomical sites.

## Figures and Tables

**Table 1:** Maternal profile during the first and second pregnancy.

	FIRST PREGNANCY									
	*n*	ND	*n*	HFD	*p*	*n*	ND	*n*	HFD	*p*
	Before Gestation					During Gestation (Two Weeks before Delivery)				
Body weight (kg)	5	24.8 ± 2.1	5	33.0 ± 2.8	0.05	5	41.6 ± 2.0	5	55.6 ± 4.0	0.01
Waist circumference (cm)	5	82.2 ± 0.9	5	90.6 ± 4.6	ns	5	103.4 ± 2.1	5	113.4 ± 3.3	0.03
Fasting glycemia (mmol/L)	5	2.87 ± 0.27	5	3.20 ± 0.32	ns	5	2.63 ± 0.48	5	2.79 ± 0.28	ns
Whole body insulin sensitivity (mg/(kg·min))	5	8.03 ± 0.61	5	6.29 ± 0.84	ns	5	7.54 ± 0.79	5	5.86 ± 0.86	ns
Liver glucose uptake (GU) (µmol/(mL·min))	5	0.13 ± 0.02	5	0.16 ± 0.01	ns	5	0.19 ± 0.03 ^#^	5	0.15 ± 0.01	ns
Duration of first gestation (days)	-	**-**	-	**-**	-	5	112 ± 1	4	111 ± 2	ns
	**SECOND PREGNANCY**									
	***n***	**ND**	***n***	**NdD**	***p***	***n***	**ND**	***n***	**NdD**	***p***
	**Before Gestation**					**During Gestation (Two Weeks before Delivery)**				
Body weight (kg)	4	48.8 ± 4.8	4	47.5 ± 3.2	ns	3	60.00 ± 2.89	4	59.75 ± 1.03	ns
Weight gain between two pregnancies (kg)	4	6.5 ± 3.2	4	−7.0 ± 2.7	0.02	-	-	-	-	-
Waist circumference (cm)	4	105.3 ± 3.5	4	102.3 ± 3.9	ns	3	118.33 ± 3.67	4	117.75 ± 2.75	ns
Fasting glycemia (mmol/L)	4	3.60 ± 0.47	4	2.60 ± 0.31	ns	3	2.60 ± 0.21	4	2.85 ± 0.21	ns
Whole body insulin sensitivity (mg/(kg·min))	4	5.12 ± 1.05	4	3.77 ± 0.61	ns	3	6.23 ± 0.68	4	6.14 ± 0.48 ^#^	ns
Liver glucose uptake (GU) (µmol/(mL·min))	4	0.16 ± 0.03	3	0.18 ± 0.01	ns	3	0.20 ± 0.03	4	0.12 ± 0.02 ^#^	0.06
Duration of second gestation (days)	-	**-**	-	**-**	-	3	109 ± 2	4	111 ± 0	ns

Values are means ± SEM. ^#^ *p* < 0.05 vs. respective group before gestation.

**Table 2:** Metabolic profile in the offspring.

FIRST PREGNANCY			
	Waist Circumference (cm)	Basal Glycemia (mmol/L)	Basal Insulinemia (mU/L)
One-week (At birth)			
ND_off_	22.9 ± 1.3 (n = 11)	6.6 ± 0.6 (n = 11)	11.4 ± 2.9 (n = 9)
HFD_off_	24.3 ± 0.8 (n = 8)	8.3 ± 0.7 ^^^ (n = 8)	22.6 ± 7.8 ^^^ (n = 3)
1-month (Infancy)			
ND_off_	37.1 ± 1.1 (n = 17)	5.3 ± 0.2 (n = 16)	11.1 ± 2.6 (n = 7)
HFD_off_	37.0 ± 0.7 (n = 20)	5.8 ± 0.4 (n = 20)	10.6 ± 3.2 (n = 5)
6-months (Early adulthood)			
ND_off_	62.2 ± 1.2 (n = 13)	3.7 ± 0.4 (n = 13)	4.6 ± 1.1 (n = 10)
HFD_off_	64.2 ± 1.6 (n = 12)	3.2 ± 0.2 (n = 12)	9.6 ± 3.8 (n = 9)
12-months (Late adulthood)			
ND_off_	75.3 ± 2.9 (n = 4)	3.4 ± 0.2 (n = 4)	2.8 ± 0.9 (n = 4)
HFD_off_	75.8 ± 4.0 (n = 4)	2.6 ± 0.9 (n = 4)	3.1 ± 2.0 (n = 3)
**SECOND PREGNANCY**			
One-week (At birth)			
ND_off_	24.9 ± 0.5 (n = 9)	7.7 ± 0.4 (n = 9)	13.7 ± 2.7 (n = 9)
NdD_off_	27.0 ± 0.4 (n = 4)	8.3 ± 0.5 (n = 4)	7.5 ± 6.1 (n = 2)
1-month (Infancy)			
ND_off_	39.5 ± 0.7 ^§^ (n = 15)	5.1 ± 0.2 (n = 15)	17.1 ± 4.2 (n = 4)
NdD_off_	39.6 ± 0.8 ^#^ (n = 11)	6.3 ± 0.5 * (n = 11)	5.0 ± 0.9 * (n = 6)
6-months (Early adulthood)			
ND_off_	71.5 ± 1.7 ^###^ (n = 8)	3.6 ± 0.3 (n = 8)	6.2 ± 1.3 (n = 7)
NdD_off_	62.8 ± 1.6 ** (n = 9)	3.8 ± 0.4 (n = 9)	3.4 ± 0.8 (n = 8)
12-months (Late adulthood)			
ND_off_	91.5 ± 0.5 ^#^ (n = 2)	2.1 ± 0.2 ^§^ (n = 2)	3.0 ± 1.8 (n = 2)
NdD_off_	74.0 ± 2.3 * (n = 3)	3.5 ± 0.7 ^^^^ (n = 3)	2.3 ± 1.7 (n = 3)

Values are means ± SEM. * *p* ≤ 0.05, ** *p* ≤ 0.01, ^^^^ *p* ≤ 0.06, ^^^ *p* < 0.1 vs. age-matched control within each pregnancy; ^#^ *p* ≤ 0.05, ^###^ *p* ≤ 0.001, ^§^ *p* < 0.8 vs. respective group in the first pregnancy.

**Table 3:** Liver histological parameters in the offspring.

		First Pregnancy								
		At One-Week			At 6-Months			At 12-Months		
	Score	ND_off_	HFD_off_	*p*	ND_off_	HFD_off_	*p*	ND_off_	HFD_off_	*p*
Microcirculation										
PV dilatation	Yes/No	100/0	100/0	ns	100/0	100/0	ns	100/0	100/0	ns
CV dilatation	Yes/No	100/0	100/0	ns	100/0	100/0	ns	100/0	100/0	ns
Sinusoid dilatation	Yes/No	36/64	43/57	ns	100/0	100/0	ns	75/25	100/0	ns
Fibrosis										
Portal	Yes/No	9/91	0/100	ns	50/50	67/33	ns	100/0	75/25	ns
Perisinusoidal	Yes/No	0/100	0/100	ns	0/100	0/100	ns	0/100	0/100	ns
Perivenous	Yes/No	0/100	0/100	ns	0/100	0/100	ns	25/75	0/100	ns
Portal inflammation	Yes/No	27/73	29/71	ns	17/83	33/67	ns	75/25	75/25	ns
Lobular damage										
Lobular inflammation	Yes/No	9/91	29/71	ns	17/83	0/100	ns	25/75	25/75	ns
Ballooning degeneration	Yes/No	36/64	71/29	ns	0/100	0/100	ns	0/100	0/100	ns
Cumulative steato-inflammatory score ^§^	NAFLD/Healthy	82/18	86/14	ns	17/83	100/0	*	0/100	50/50	ns
		**Second pregnancy**								
		**ND_off_**	**NdD_off_**		**ND_off_**	**NdD_off_**		**ND_off_**	**NdD_off_**	
Microcirculation										
PV dilatation	Yes/No	100/0	100/0	ns	100/0	100/0	ns	100/0	100/0	ns
CV dilatation	Yes/No	89/11	100/0	ns	100/0	100/0	ns	50/50	100/0	ns
Sinusoid dilatation	Yes/No	11/89	0/100	ns	80/20	60/40	ns	50/50	100/0	ns
Fibrosis										
Portal	Yes/No	0/100	0/100	ns	0/100	40/60	ns	50/50	33/67	ns
Perisinusoidal	Yes/No	0/100	0/100	ns	0/100	0/100	ns	0/100	0/100	ns
Perivenous	Yes/No	0/100	0/100	ns	0/100	0/100	ns	0/100	0/100	ns
Portal inflammation	Yes/No	56/44	33/67	ns	20/80	80/20	ns	50/50	33/67	ns
Lobular damage										
Lobular inflammation	Yes/No	0/100	33/67	^#^	0/100	0/100	ns	0/100	0/100	ns
Ballooning degeneration	Yes/No	0/100	0/100	ns	0/100	0/100	ns	0/100	0/100	ns
Cumulative steato-inflammatory score ^§^	NAFLD/Healthy	0/100	0/100	ns	40/60	40/60	ns	50/50	100/0	ns

Categorical scores indicate the number percentage (%) of animals in each group presenting with (=Yes) or without (=No) the abnormality, and healthy or unhealthy (NAFLD) condition; ^§^ = Cumulative steato-inflammatory score was determined by the sum of steatosis grades (1–3), lobular inflammation (1–3), and ballooning grades (1–2), reflecting progressive disease severity stages. * *p* = 0.045, ^#^ *p* = 0.07 vs. age-matched control within each pregnancy.

## Data Availability

The data presented in this study are available on request from the corresponding author, as they have not yet been uploaded in a public database.
